# E-Scooter and Bicycle Healthcare Utilization in U.S. Emergency Departments: Examined Through Mode-Specific Diagnostic Codes

**DOI:** 10.3390/ijerph23080972

**Published:** 2026-07-28

**Authors:** Leomar White, Christiana Zimmer, Jason Jackman, Karen Liller, Jason L. Salemi

**Affiliations:** 1 Department of Epidemiology, College of Public Health, University of South Florida, Tampa, FL 33612, USA; jsalemi@usf.edu; 2 Department of Community Health Sciences, College of Public Health, University of South Florida, Tampa, FL 33612, USA; cmzimmer@usf.edu (C.Z.); kliller@usf.edu (K.L.); 3 City of Tampa Mobility Department, Tampa, FL 33602, USA; jason.jackman@tampagov.net

**Keywords:** electric scooter, pedal bicycle, injury, emergency department, healthcare resource utilization, motor vehicle collision, effect modification, causal inference, Nationwide Emergency Department Sample, ICD-10-CM codes

## Abstract

**Highlights:**

**Public health relevance—How does this work relate to a public health issue?**

- E-scooter injuries are increasing nationwide, but precise national estimates of health resource utilization are lacking.
- This study uses newly available e-scooter-specific ICD-10-CM codes to fill a critical gap in injury surveillance.

**Public health significance—Why is this work of significance to public health?**

- Motor vehicle involvement significantly increases hospital admission odds for e-scooter riders compared to bicyclists.
- E-scooter and bicycle crashes produce distinct injury patterns requiring different prevention approaches, with riders under 18 disproportionately affected.

**Public health implications—What are the key implications or messages for practitioners, policy makers and/or researchers in public health?**

- There are no federal safety policies regulating the use of e-scooters, which is contrasted by decades of bicycle-related policy.
- These findings may help inform future transportation safety initiatives and identify areas where additional evaluation of infrastructure and injury-prevention strategies may be warranted.

**Abstract:**

Few national comparisons exist on healthcare utilization among e-scooter users, and prior research was limited by the absence of e-scooter-specific ICD-10-CM codes before 2021. Using newly implemented codes, this cross-sectional study compared emergency department disposition and injury patterns between e-scooter riders and bicyclists using the 2021 Nationwide Emergency Department Sample (*n* = 239,457 weighted encounters). Multivariable logistic regression adjusted for demographic, geographic, and temporal confounders, with interaction terms for insurance status, substance use, and motor vehicle involvement. E-scooter riders were more often female (46.5% vs. 24.7%) and under age 18 (47.6% vs. 37.4%) than bicyclists. Overall adjusted odds of admission or transfer did not differ significantly between groups (OR = 1.03; 95% CI: 0.86–1.23). Motor vehicle involvement significantly modified this relationship, with e-scooter riders showing higher odds of admission (OR = 1.67; 95% CI: 1.12–2.48). No significant effect modification was detected for insurance status or substance use. E-scooter riders more frequently sustained distal extremity fractures consistent with low-energy falls, while bicyclists experienced more proximal fractures and internal trauma. Updated ICD-10-CM codes improve e-scooter injury surveillance, and motor vehicle involvement represents a priority target for mode-specific prevention and infrastructure investment.

## 1. Introduction

The rapid growth in the use of micromobility, particularly electric scooters (e-scooters), has reshaped short-distance travel in U.S. cities. Micromobility refers to small, low-speed, human- or electric-powered transportation devices, including conventional bicycles, electric bicycles (e-bikes), and electric scooters (e-scooters). Operating characteristics, including typical travel speeds, vary by vehicle type and local regulations. E-scooters are small, low-speed, electrically powered devices used for personal or shared short-distance urban travel, commonly rented through smartphone applications or self-service kiosks [1,2]. In 2021, over 52 million trips were taken on shared e-scooters, with ridership approaching bicycle levels in some areas [3,4]. As injury counts increase with ridership, understanding how healthcare use and emergency department (ED) patterns for e-scooter injuries differ from those for traditional modes like bicycles is important for allocating emergency resources and guiding prevention efforts.

The U.S. Consumer Product Safety Commission (CPSC) reported a 66% increase in e-scooter-related ED encounters from 2020 to 2021, with injuries continuing to trend upward into 2022 [5,6]. In contrast, bicycle-related injuries remained relatively stable during this period, following a brief pandemic-related spike in 2020 [7]. Because both groups experience minimal physical protection and are exposed to urban traffic, direct comparisons between e-scooter riders and bicyclists are essential for informing injury-prevention strategies and transportation policy. Bicyclists have benefited from decades of dedicated lane infrastructure and traffic-calming advocacy. However, e-scooter riders frequently operate in a regulatory gray area, choosing between sidewalks and car lanes. Many states and local governments have stepped in to establish their own regulations, with shared e-scooter programs often being highly regulated at the local level [8,9].

E-scooters are not a homogeneous category; shared fleet scooters differ from privately owned scooters in speed, weight, maintenance quality, and rider experience. E-scooter-related injuries range from minor abrasions to severe head trauma and multi-system injuries. Extremity fractures are the most common injuries among both e-scooter riders and bicyclists, while head injuries such as concussions and intracranial hemorrhages are strongly associated with hospital admission and mortality [10,11,12,13,14]. Mechanisms of injury differ by mode: e-scooter crashes often result from low-height falls or collisions with fixed objects, whereas bicyclist injuries more frequently involve motor vehicles or occur in rural areas, both of which are linked to greater health resource utilization [15,16]. Substance use, particularly alcohol and drugs, further elevates the risk of hospitalization and mortality across both groups [17].

Beyond clinical and crash-level factors, insurance status is a well-established determinant of healthcare utilization following traumatic injury in the United States. Prior research has demonstrated that uninsured and underinsured patients face financial barriers that influence ED disposition decisions, with self-pay patients more likely to be discharged home than transferred or admitted compared with insured patients, even after accounting for injury severity [18,19,20]. Although previous studies have demonstrated that insurance status independently influences emergency department disposition following traumatic injury, whether this association differs according to transportation mode has not been examined. Accordingly, we evaluated insurance status as an exploratory effect modifier to determine whether the association between transportation mode and emergency department disposition varied across insurance categories.

A major limitation in earlier research has been the lack of specific ICD-10-CM codes for electric standing scooters before 2021. As a result, previous studies relied on proxy definitions that filtered cases using broader motorized scooter codes and were often restricted to patients under age 64 without documented neurological disorders. This excluded motorized wheelchairs and other mobility aids. This approach, while practical, introduced potential misclassification and limited the precision of exposure measurement. This study builds on existing literature by using the newly introduced e-scooter-specific codes (effective October 2020) to more precisely capture relevant injury encounters, enabling a robust comparison with bicycle-related injuries.

### 1.1. Background Study

We leveraged data from the 2021 Healthcare Cost and Utilization Project (HCUP) Nationwide Emergency Department Sample (NEDS), the largest publicly available all-payer ED database in the United States [21], to analyze these updated codes at the national level. NEDS captures encounters using standardized ICD-10-CM diagnostic codes, enabling more granular characterization of injuries and richer covariate adjustment. These features make NEDS particularly well-suited for comparative micromobility health resource utilization and address a key limitation of prior studies that relied on smaller, region-specific, or less precisely coded datasets. Because this study draws on ED-based data, findings reflect injury and utilization patterns among those who sought emergency care rather than absolute riding risk. Trip- or mile-level exposure data are unavailable in this dataset, and no inference is made regarding which mode is categorically more dangerous to ride.

Understanding how these updated ICD-10-CM diagnostic codes fit within broader injury trends is critical for interpreting the generalizability of 2021 findings. Evidence from this period suggests that e-scooter encounters broadly aligned with sustained increases rather than representing anomalies. The CPSC reported that fractures and head and neck injuries were the most common diagnoses, consistent with pre-pandemic trends [5], and shared micromobility injuries continued to rise by 21% in 2022, indicating that 2021 was part of an ongoing upward trajectory [6]. Peer-reviewed studies examining injury mechanisms and demographic patterns during this period support the relative stability of key factors, including motor vehicle involvement and age-based outcome disparities [11,22]. Prior research on micromobility-related crashes presents evidence that vehicle type, crash configuration, and rider characteristics exhibit comparably stable effects across periods and geographic settings, further supporting the representativeness of data from this window [23,24].

However, the 2021 data were collected amid ongoing pandemic-related disruptions, which should be acknowledged with due consideration. Changes in travel behavior, reduced motor vehicle traffic volumes, and altered healthcare-seeking patterns during pandemic recovery may have introduced distortions that injury mechanism trends alone do not fully capture [25,26]. The micromobility landscape has also continued to evolve. Shared fleet e-scooters increasingly feature geofenced speed limiters and improved braking systems, and urban infrastructure has adapted in many cities since 2021 [27]. Recent research shows that speed, road environment, and rider behavior are key factors in the severity of micromobility crashes across years and settings [28,29,30]. While injury mechanisms are stable, the risk profile of e-scooter users may have changed as technology, regulation, and infrastructure have evolved.

### 1.2. Present Study

This study addresses key gaps in the micromobility injury literature by using the 2021 NEDS to compare health resource utilization following e-scooter and bicycle-related injury. Specifically, we aim to assess whether e-scooter injuries are more likely to result in hospital admission or transfer than bicycle-related injuries. A secondary aim is to evaluate whether this association differs by motor vehicle involvement and substance use, and to explore whether primary insurance status modifies this relationship. Insurance status was evaluated as an exploratory, hypothesis-generating effect modifier because differences in healthcare access, reimbursement, and disposition practices may influence admission decisions differently across transportation modes, although this relationship has not previously been examined. The final aim is to describe the types of injuries sustained by e-scooter riders and bicyclists, to inform more targeted prevention efforts.

We hypothesized that, after accounting for demographic and injury-related factors, e-scooter riders would have higher adjusted odds of hospitalization or transfer compared to bicyclists, and that these odds would be elevated in the presence of motor vehicle involvement or documented substance use. By examining the relationship between device type and ED disposition, this study aims to inform clinical triage protocols and to support the development of equitable, data-driven injury-prevention strategies for micromobility users. Because this study is based on emergency department encounters, the findings reflect healthcare utilization and injury patterns among patients who sought emergency care and should not be interpreted as estimates of injury risk among all bicycle or e-scooter users.

## 2. Methods

### 2.1. Data Source

NEDS, maintained by the Agency for Healthcare Research and Quality (AHRQ), contains only de-identified, publicly available data, making this study exempt from IRB review under 45 CFR §46.104(d) [31]. The study was restricted to 2021 because it was the first full calendar year in which e-scooter-specific ICD-10-CM external cause codes were available and consistently implemented nationwide, enabling more precise identification of e-scooter-related injuries than in prior years. According to [32], the 2021 NEDS was constructed from data contributed by 40 states and the District of Columbia, sampling 993 hospital-owned EDs and capturing approximately 30 million ED visits, which, after applying discharge weights, expanded to a nationally representative estimate of approximately 127 million visits. The participating states account for 84.0% of the total U.S. resident population and 83.4% of all U.S. ED visits. The stratified sampling design ensures representation across five key hospital characteristics: geographic region, trauma center designation, urban-rural location, teaching status, and hospital ownership, producing a nationally representative cross-section of the ED landscape rather than a convenience or regional sample [32].

### 2.2. Study Population

**Figure 1:**
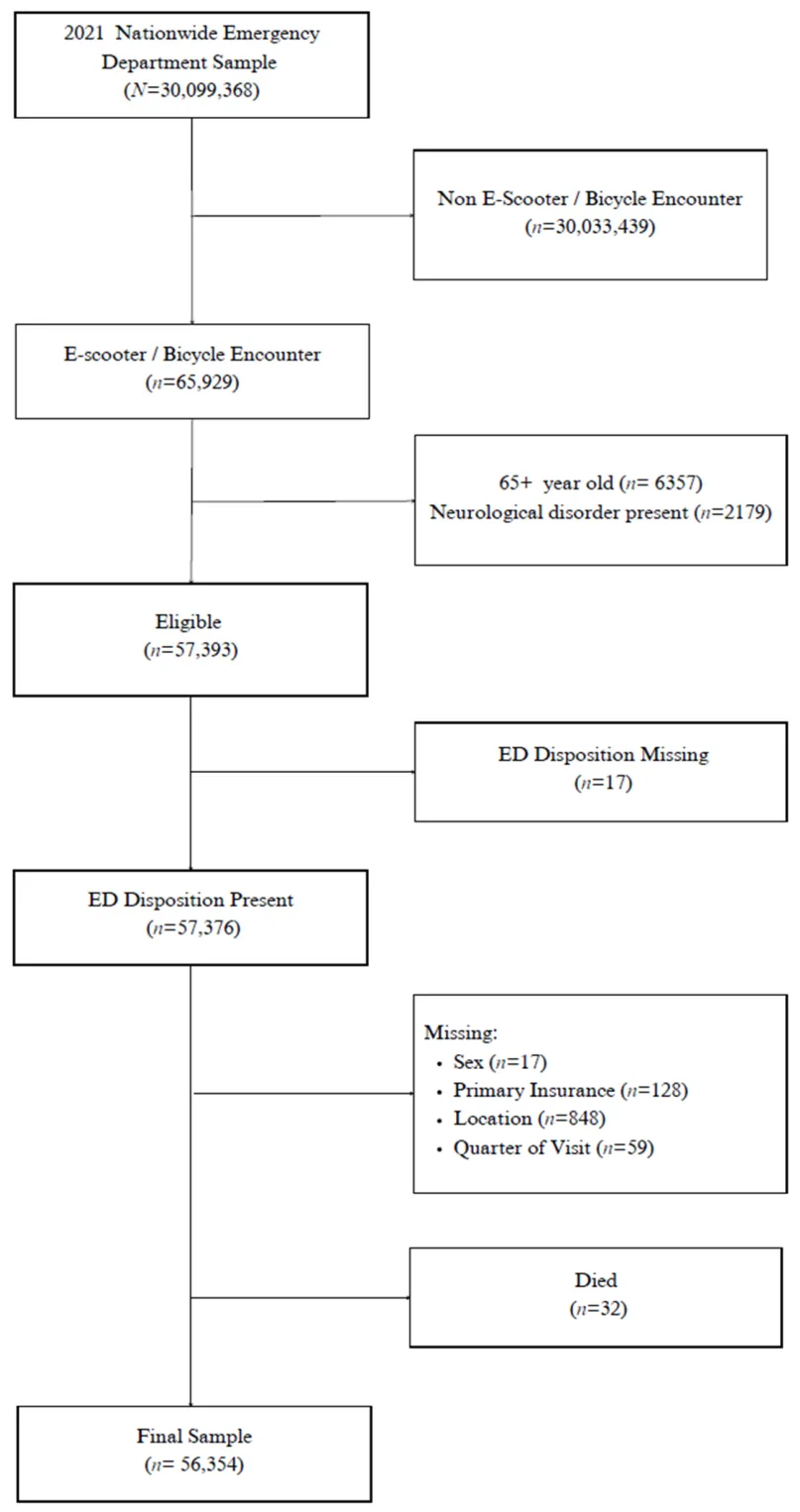
Study Population Flowchart and Analytic Sample Selection. A flow diagram showing the listwise deletion steps for the analytic sample. Some encounters were excluded due to missing data in one or more covariates. The counts shown represent the number of records excluded for each covariate, but they should not be summed up because a single encounter may be missing multiple covariates.

The study population included ED visits for individuals younger than 65 with no neurological disorders (G00-99) and with ICD-10-CM external cause codes indicating a bicycle (V10–V19) or e-scooter (V0084-, V01031-, V06131, etc.) injury. A complete list of codes is provided in Supplemental Table S1. These inclusion criteria minimize measurement bias by improving the identification of true e-scooter injuries. Individuals aged 65 years or older or with neurological disorders were excluded because they may use motorized mobility aids that could be misclassified as e-scooters. Visits with missing data on primary exposures, ED disposition, and key covariates were also excluded. After applying these criteria, the analytic sample was reduced from 65,929 e-scooter and bicycle-related encounters to 56,354 (see Figure 1).

### 2.3. Variables

**Figure 2:**
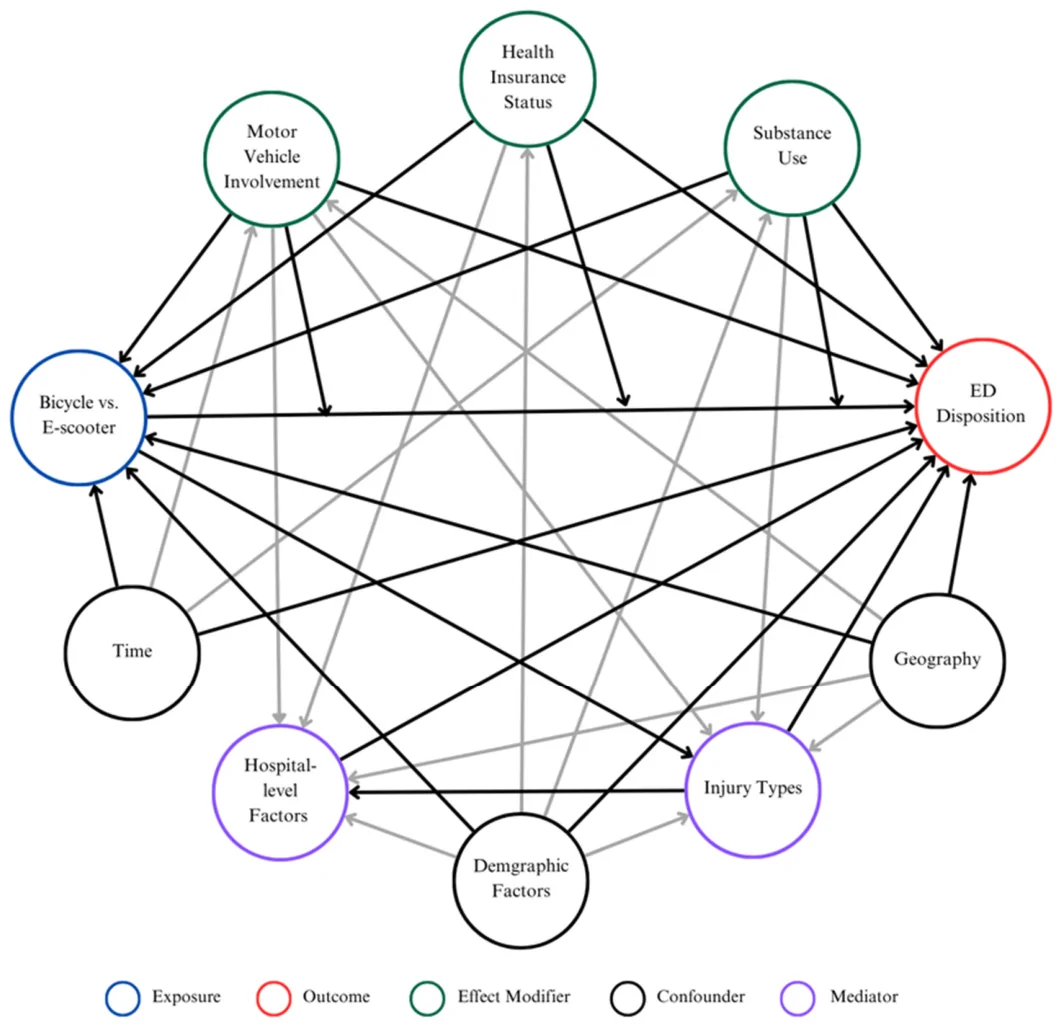
Directed Acyclic Graph. A directed acyclic graph showing the hypothesized relationships between each study variable. Black arrows indicate primary causal paths of interest. Gray arrows indicate additional hypothesized relationships.

A directed acyclic graph (DAG) was developed to depict hypothesized relationships among study variables and identify a minimally sufficient set of confounders (see Figure 2). The outcome was ED disposition, initially categorized: (1) death in the ED, (2) admitted or transferred to another hospital, and (3) treated and released. Because few records indicated death following ED presentation (*n* = 32, see Figure 1), these records were excluded, and the outcome was analyzed as a binary variable: admitted or transferred (coded 1) versus treated and released (coded 0). Admission and transfer were combined because both represent escalation of care beyond ED treatment and release, reflecting substantially greater acute healthcare resource utilization than discharge home. Although ED disposition represents only one dimension of healthcare utilization, it serves as an important indicator of acute healthcare resource use following injury.

The primary exposure was the mode of transportation (conventional bicyclist vs. e-scooter rider). Effect modifiers included motor vehicle involvement, substance use related to alcohol, and primary insurance status, categorized as uninsured or underinsured, public, and private (see Supplemental Table S1). Confounders were identified using the DAG, the existing literature, and the availability of variables in the dataset. They included demographic factors (age, biological sex, and race), geographic variables (urban versus rural residence and hospital region), and temporal variables (weekend and quarter of the visit). Quarter of visit was used in place of month because certain states suppress month-level data in NEDS to protect patient confidentiality, consistent with HCUP data use guidelines [33].

Hospital-level characteristics, including teaching status, ownership, and trauma level, were initially considered as potential confounders but were reclassified as mediators following DAG review. These variables were judged to lie on the causal pathway between mode of transportation and ED disposition, rather than to serve as independent determinants of both. Specifically, the type of hospital a patient presents to is influenced by the nature and circumstances of the injury, including the mode of transport involved. This directly shapes the likelihood of admission or transfer through institutional capacity, triage protocols, and resource availability. Adjusting for these variables in the primary model would therefore block part of the causal pathway of interest and yield only an estimate of the direct effect, excluding the portion of the effect that operates through the hospital context. To preserve an estimate of the total effect of device type on ED disposition, hospital-level characteristics were excluded from the adjusted model. This decision is consistent with established principles of causal inference in observational research and is reflected in the DAG structure presented in Figure 2. Still, alternative causal structures are possible, and hospital characteristics may reasonably be conceptualized as confounders, mediators, or selection variables depending on the research question and causal estimand.

Injury type was conceptualized as a potential mediator between mode of transportation and ED disposition and was therefore excluded from multivariable models to avoid overadjustment bias. To provide clinical context, injury types were summarized descriptively and categorized as minor head injuries, intracranial injuries, fractures by anatomical location, dislocations, sprains, intra-abdominal or intrathoracic injuries, and lacerations. The corresponding ICD-10-CM codes for each category are listed in Supplemental Table S1.

### 2.4. Statistical Analysis

All Survey-weighted analyses were conducted in R (version 4.4.1) using the survey package following HCUP recommendations [32]. The survey design object incorporated the NEDS discharge weights (DISCWT), hospital-based primary sampling units (HOSP_ED), and the NEDS stratification variable (NEDS_STRATUM). Variance estimates were obtained using Taylor series linearization. Strata containing a single primary sampling unit were handled using the survey package’s conservative adjustment for singleton PSUs. Descriptive statistics were generated using survey-weighted frequencies and proportions, with differences assessed using Rao-Scott adjusted chi-square tests. The association between mode of transportation (bicycle vs. e-scooter) and ED disposition was estimated using survey-weighted binary logistic regression. The dependent variable distinguished between admitted or transferred (1) versus treated and released (0). In addition to a crude (unadjusted model), an adjusted model controlled for a minimally sufficient set of confounders, including demographic, geographic, and temporal factors.

Effect modification was assessed on the multiplicative scale by including interaction terms between transportation mode and each pre-specified modifier: (1) motor vehicle involvement (yes vs. no), (2) substance use (yes vs. no), and (3) primary insurance type (private, public vs. uninsured or under-insured, the referent). Wald tests were used to assess the statistical significance of interaction terms. If a significant interaction was detected, stratum-specific adjusted models were fit to estimate the association between transportation mode and the outcome within levels of the effect modifier. Effect modification was evaluated on the multiplicative scale because the primary objective was to determine whether the relative association between transportation mode and emergency department disposition differed across prespecified subgroups. Additive interaction measures quantify the excess absolute risk attributable to joint exposures and are useful for a range of scientific questions, particularly those focused on population impact. Because the present study was designed to evaluate heterogeneity in relative associations rather than excess absolute risk, interaction was assessed on the multiplicative scale.

Substance use was initially categorized by type: no substance use, alcohol only, drugs only, and both alcohol and drugs. However, the alcohol-only category contained suppressed unweighted cell counts in both exposure groups, falling at or below the HCUP Data Use Agreement threshold of 10 or fewer observations, which prohibits release of cell-level data at that frequency. Weighted frequencies are reported descriptively in Table 1, but due to the underlying sparsity of observed cases, substance use was collapsed into a binary indicator (any substance use vs. none) for multivariable modeling to ensure stable effect estimates and comply with HCUP data use guidelines [32]. Adjusted odds ratios and 95% confidence intervals were reported for all effect estimates. All analyses excluded records with missing data using listwise deletion, given the low proportion of missingness across covariates.

## 3. Results

### 3.1. Sample Characteristics

The weighted analytic sample represented 239,457 ED visits, of which 20,988 (8.8%) involved e-scooter riders (Table 1). Most encounters were treated and released, with similar proportions between bicyclists (91.7%) and e-scooter riders (93.4%; *p* = 0.007). Several demographic and clinical characteristics differed significantly between groups. For example, minors accounted for a larger share of e-scooter patients than of bicyclists (47.6% vs. 37.4%; *p* < 0.001). A higher proportion of e-scooter patients were biological females (46.5% vs. 24.7%; *p* < 0.001) and covered by public insurance (43.5% vs. 37.2%; *p* < 0.001), though bicyclists had slightly higher rates of being uninsured or underinsured (13.2% vs. 15.2%; *p* < 0.001).

Bicyclists were more frequently involved in collisions with motor vehicles (16.9% vs. 7.7%; *p* < 0.001), while substance use rates were nearly identical across both groups. Although the distributions of race and location were broadly similar, e-scooter users were more likely to reside in the lowest household income quartile (29.7% vs. 22.8%; *p* < 0.001), whereas bicyclists were more often in the highest household income quartile (29.5% vs. 22.6%; *p* < 0.001). Slightly more e-scooter visits occurred on weekends (36.9% vs. 33.8%; *p* < 0.001). E-scooter visits peaked in quarter 2 (29.8%) while bicyclists peaked in quarter 3 (34.2%). Regionally, encounters for both groups were more concentrated in the West (30.8% [e-scooter] and 34.6% [bicycle]). Refer to Table 1 for more information.

### 3.2. Injury Profile

Injury patterns differed by mode of transportation (Table 2). Because patients may sustain multiple injuries, column percentages may exceed one hundred. Lacerations were the most common injury overall (21.1%) and were significantly more frequent among bicyclists (21.6%) than e-scooter riders (16.2%, *p* < 0.001). Distal upper extremity fractures were the second most common and occurred more often among e-scooter riders (23.0% vs. 14.0%, *p* < 0.001). E-scooter riders also had slightly higher rates of distal lower extremity fractures (6.0% vs. 4.7%, *p* = 0.001) and sprains (7.8% vs. 6.7%, *p* = 0.007). In contrast, bicyclists sustained significantly more proximal upper extremity fractures (9.3% vs. 7.6%, *p* < 0.001) and proximal lower extremity fractures (1.1% vs. 0.8%, *p* = 0.040). Bicyclists were also more likely to experience severe injuries to the trunk and internal organs. They had higher rates of fractures to the ribs, sternum, and thoracic spine (5.0% vs. 1.8%, *p* < 0.001), as well as pelvic and lumbar spine fractures (1.5% vs. 0.7%, *p* < 0.001). Dislocations (3.8% vs. 2.3%, *p* < 0.001) and intra-abdominal or intrathoracic injuries (3.4% vs. 1.4%, *p* < 0.001) were also more common among bicyclists. Despite these differences, the two groups had similar rates of minor head injuries, intracranial injuries, and fractures of the skull or facial bones (*p* > 0.05) (see Table 2).

### 3.3. Multivariable Binomial Logistic Regression

In the unadjusted model, e-scooter riders had significantly lower odds of hospital admission or transfer compared with bicyclists (OR = 0.78, 95% CI: 0.65–0.94). However, after adjusting for demographic, geographic, and temporal confounders, the association remained non-significant (adjusted OR = 1.03, 95% CI: 0.86–1.23). A statistically significant multiplicative interaction was observed between transportation mode and motor vehicle involvement (*p* = 0.002). Stratified analyses showed that among encounters involving motor vehicles, e-scooter riders had significantly higher odds of hospital admission or transfer compared with bicyclists (adjusted OR = 1.67, 95% CI: 1.12–2.48). No significant interactions were found for insurance status (*p* = 0.326) or substance use (*p* = 0.402). Full model results are presented in Table 3.

## 4. Discussion

### 4.1. Main Findings

Despite the rapid growth of e-scooter ridership in U.S. cities [3,5], this study found no statistically significant difference in the odds of hospital admission or transfer between e-scooter riders and bicyclists after adjusting for demographic, temporal, and geographic factors. These results suggest that, within the constraints of the NEDS dataset, overall health resource utilization as proxied by ED disposition appears comparable between the two modes. That said, ED disposition is a coarse measure that does not distinguish between short-stay observation, ICU admission, and hospital resource intensity, thereby limiting its sensitivity to gradations in injury burden. Because injury severity was conceptualized as a mediator rather than a confounder, the adjusted models estimate the total association between transportation mode and emergency department disposition. Therefore, these findings should not be interpreted as representing severity-independent effects. Although hospital admission and interhospital transfer represent distinct clinical pathways, both indicate escalation of care beyond emergency department treatment and release. However, transfer may reflect hospital capability rather than injury severity, introducing heterogeneity that this study cannot fully disentangle. Future studies with larger sample sizes may be able to evaluate these outcomes separately. The difference between the crude and adjusted odds ratios should not be interpreted solely as evidence of confounding because odds ratios are non-collapsible. Accordingly, our modeling strategy emphasized identifying a minimally sufficient adjustment set using a directed acyclic graph rather than attributing changes in nested odds ratio estimates to individual covariates.

The comparability of ED utilization observed here is further supported by the national scope of the analysis. Prior micromobility injury studies have largely relied on single-site trauma registries, regional datasets, or NEISS, which sample approximately 100 hospitals and use consumer product injury codes rather than standardized ICD-10-CM classifications. The present study draws on a survey-weighted, nationally representative sample stratified across hospital type, geographic region, and trauma designation, producing estimates that reflect the full diversity of ED settings rather than a particular institution or region. The null finding for overall admission odds is therefore unlikely to reflect idiosyncratic features of a specific hospital or patient population. The motor vehicle interaction finding, which held across this diverse national sample, carries corresponding generalizability. Together, these features strengthen confidence that the observed patterns represent meaningful national-level differences in micromobility injury utilization rather than sampling artifacts.

### 4.2. Multiplicative Interaction

Motor vehicle involvement was relatively uncommon in this sample, yet it produced the strongest and most precise effect estimate in the analysis. When a motor vehicle was involved, e-scooter riders had significantly higher odds of hospital admission or transfer compared to bicyclists (adjusted OR = 1.67, 95% CI: 1.12–2.48), a finding that held after adjustment for demographic, temporal, and geographic factors. This pattern is consistent with prior evidence documenting the disproportionate severity of motor vehicle interactions for micromobility users. Ref. [34] using police report data, estimated that approximately 10% of micromobility crashes involving a motor vehicle result in fatal outcomes, while [14], analyzing NEISS data, found that approximately 14% of e-scooter crashes involving motor vehicles resulted in hospital admissions between 2014 and 2019. Although direct numerical comparisons are not appropriate because previous studies reported descriptive admission proportions rather than adjusted effect estimates, our findings are directionally consistent with prior literature demonstrating greater clinical severity among crashes involving motor vehicles. Taken together, the evidence suggests that motor vehicle involvement represents a high-leverage point for intervention, warranting clinical and policy attention for e-scooter riders.

E-scooter riders stand upright on a narrow platform with no frame structure to absorb impact energy, leaving them with less postural stability and fewer mechanical buffers at the moment of collision compared to seated bicyclists [35,36]. Additionally, e-scooters are more frequently operated in mixed-traffic environments, on roads without dedicated infrastructure, and by riders with less experience navigating interactions with motor vehicles, all of which may amplify crash severity when a motor vehicle is involved [37,38,39]. Prior computational modeling has similarly suggested that standing posture and lower platform height increase head-ground impact forces during sudden deceleration events [36,40]. Several factors proposed in previous studies, including rider posture, vehicle design, infrastructure, and riding experience, may contribute to the observed differences; however, these factors were not measured in NEDS and therefore remain plausible explanations informed by prior literature rather than direct inferences from the present study.

Crashes involving motor vehicles serve as a critical point for intervention. Targeted strategies might include infrastructure solutions such as protected scooter lanes that physically separate riders from vehicle traffic, speed-limiting geofencing in high-traffic corridors, and EMS triage protocols that explicitly account for mode of transport and motor vehicle involvement as markers of elevated admission risk. Stronger data linkage between ED records, pre-hospital EMS data, and police crash reports would substantially improve the ability to characterize this subgroup and evaluate the impact of such interventions over time.

No significant effect modification was detected for insurance status or substance use in this analysis. For insurance status, the absence of significant interaction does not preclude the possibility that access barriers influence care-seeking behavior prior to ED presentation, a pathway that NEDS, by design, cannot capture. Future studies linking ED records with pre-hospital and claims data would be better positioned to evaluate whether insurance status shapes utilization patterns upstream of the ED encounter.

For substance use, the null interaction finding should be interpreted cautiously, given that substance use in NEDS is identified through diagnostic codes rather than toxicology results, increasing the likelihood of underreporting and potentially attenuating any true effect. Despite the absence of significant effect modification in the present analysis, prior research suggests that substance use remains a clinically meaningful factor in micromobility injury outcomes. Ref. [17], using National Inpatient Sample data, reported an eightfold increase in substance use-related e-scooter admissions between 2016 and 2021, with affected cases exhibiting higher odds of traumatic brain injury, perioperative complications, and increased hospitalization costs compared to e-scooter patients without substance use. These findings suggest that while substance use may not significantly modify the relationship between device type and ED disposition, it remains an important determinant of downstream resource intensity and clinical trajectory once admitted. This warrants continued attention to micromobility injury surveillance and prevention efforts.

### 4.3. Injury Profiles

Fatalities were rare within ED records in our analytic sample, suggesting that most fatal micromobility events occur at the scene rather than after hospital presentation [34]. This limitation, together with the exclusion of pre-hospital data, may underestimate the full burden of high-severity e-scooter crashes and introduce survival bias that could artificially homogenize the risk profiles of those who do present to the ED. Among those who survive to admission, however, evidence suggests that clinical trajectories may still differ by mode. Ref. [15], using National Inpatient Sample data to compare post-admission outcomes between pedal bicyclists and e-scooter riders, found that hospitalized e-scooter patients had lower adjusted odds of in-hospital mortality compared to hospitalized bicyclists, suggesting that while admission rates may be comparable across modes, bicyclists who are hospitalized may sustain more severe injuries overall.

Given the distinct injury distributions, we hypothesized that e-scooter and bicycle crashes exhibit different biomechanical profiles. Although lacerations were the most common injury across all ED encounters, they occurred significantly more often among bicyclists (21.6% vs. 16.2%, *p* < 0.001). In contrast, e-scooter riders had a notably higher rate of distal upper-extremity fractures (23.0% vs. 14.0%, *p* < 0.001), along with higher rates of distal lower-extremity fractures and sprains. These findings align with those of [12,15], who also identified distal extremity fractures—particularly of the wrist and ankle—as common among e-scooter riders. These patterns are consistent with the hypothesis that low-energy falls involve riders attempting to break their fall with their hands or feet, especially on unstable or unfamiliar terrain. This understanding is also suggested by the biomechanical literature on standing posture and e-scooter falls [36,40,41].

Bicyclists exhibited a broader distribution of musculoskeletal injuries, with significantly more proximal upper and lower extremity fractures, and were more likely to sustain severe internal trauma. Rib, sternum, and thoracic spine fractures were over twice as common in bicyclists as in e-scooter riders (5.0% vs. 1.8%, *p* < 0.001), as were pelvic and lumbar spine fractures (1.5% vs. 0.7%, *p* < 0.001). Bicyclists also had significantly higher rates of dislocation and intra-abdominal or intrathoracic injuries. These distributions are consistent with the hypothesis that bicycle crashes may involve greater energy transfer or different impact mechanics [42,43]. Ref. [11] similarly found that bicyclists had a higher likelihood of hospital admission and experienced a broader range of serious injuries than e-scooter riders, despite similar patterns of head trauma.

Both groups had comparable rates of minor head injuries, intracranial injuries, and skull or facial fractures (*p* > 0.05), a finding that masks an important prevention gap. Ref. [44] documented a national rise in e-scooter-related head trauma while simultaneously noting that helmet use remained extremely low among injured riders. This is particularly consequential given the established evidence that helmets prevent over 80% of serious head and brain injuries in bicyclists, with the protective effect especially pronounced in crashes involving motor vehicles [45]. Although NEDS does not capture helmet use, the comparable head injury rates observed here between e-scooter riders and bicyclists, combined with evidence that e-scooter riders are far less likely to wear helmets, suggest that the true head injury burden among e-scooter riders may be disproportionately preventable. Ref. [22] similarly emphasized that the frequency of head and extremity injuries among e-scooter riders remains a public health concern, particularly in shared-use urban settings where helmet use norms are not yet established.

These findings suggest that e-scooter injuries tend to involve extremities, and prior research indicates that such injuries are common in low-velocity falls [12,15,24]. In contrast, bicycle injuries more often involve high-severity impacts, internal trauma, and broader musculoskeletal involvement, likely due to variations in body positioning and fall mechanics [11,30,42,43]. Unlike bicyclists, e-scooter riders stand upright with their legs positioned in front of the device, allowing them to instinctively brace with their arms and legs during a fall. These distinctions suggest that mode-specific injury-prevention strategies may be warranted, though the present cross-sectional design cannot determine which interventions would be most effective. Strategies worth evaluating for e-scooter riders might include helmet promotions, fall-prevention training, and wrist and ankle protection, while bicyclist safety efforts may benefit from infrastructure improvements that reduce exposure to high-impact collisions. See Table 2 for detailed injury profiles. These findings may offer useful context for EMS protocols, particularly in guiding initial assessments when the mode of transport is known.

### 4.4. Strengths and Limitations

This study presents a novel approach to assessing the relationship between micromobility device type and ED disposition using standardized ICD-10-CM diagnostic codes within a nationally representative dataset. A key methodological contribution is the explicit use of a DAG to inform variable selection, distinguishing confounders from mediators and reducing the risk of overadjustment. This study is the first known use of the NEDS to compare injury outcomes between e-scooter riders and bicyclists using standardized national data. It is the first to examine insurance status as an exploratory effect modifier in this population. By leveraging a large, survey-weighted, nationally representative sample, this study improves generalizability over previous work based on smaller, regional, or convenience-based datasets.

One strength of the study lies in its use of specific ICD-10-CM codes to identify e-scooter injuries, which likely improved the precision of case identification relative to earlier studies that relied on broader proxy definitions. The lower prevalence of e-scooter injuries observed here, compared with prior literature, suggests that earlier work may have overestimated injury rates due to non-differential misclassification. Alternatively, hospital coders may still be using outdated or non-specific codes to classify standing electric scooters, which could introduce differential misclassification of exposure and bias effect estimates. Future studies should reassess previously reported associations using the newer, more specific codes to evaluate the extent of this potential bias.

Nonetheless, several limitations should be considered. First, hospital-level characteristics, including teaching status, ownership, and trauma level, were classified as mediators rather than confounders because our objective was to estimate the total association between transportation mode and emergency department disposition rather than an effect independent of downstream hospital factors. We recognize that hospital characteristics could reasonably be conceptualized as confounders, mediators, or selection variables under alternative causal structures, and that the DAG in Figure 2 reflects one plausible causal ordering rather than a definitively established one. A research question focused on the direct effect of device type, net of hospital context, would call for a different adjustment strategy and could yield different estimates; if hospital characteristics function even partly as confounders, the associations reported here, including the motor vehicle interaction effect, may differ from the direct effect of device type net of hospital context.

Second, because the NEDS captures only patients who present to an ED, the study is inherently conditioned on ED survival. This design excludes pre-hospital fatalities and riders with injuries too minor to seek care, both of which may differ systematically between e-scooter riders and bicyclists. If the kinematic differences between modes produce differential rates of at-scene fatalities or subclinical injuries, comparing only ED survivors may artificially homogenize risk profiles and contribute to the null finding observed for the primary outcome. This survival bias should be considered when interpreting all findings.

Third, the analytic sample was reduced from 65,929 eligible encounters to 56,354 after removing records with missing values on ED disposition, sex, primary insurance, patient location, and quarter of visit. Missing data were limited to these five variables for the purposes of case exclusion. Household income quartile and hospital trauma level also contained missing values due to suppressed or unreported data in the 2021 NEDS; however, because neither variable was included in the adjusted regression model, records with missing values on these variables were retained in the analytic sample, and the missing counts are reported in Table 1. Race also contained an unknown category, which was retained as a separate level in the regression model given its non-trivial size rather than being excluded via listwise deletion. A comparison of included and excluded cases revealed no meaningful differences in exposure distribution: e-scooter and bicycle encounters comprised 8.8% and 91% of the included sample and 3.8% and 96% of the excluded sample, respectively. Similarly, the distribution of insurance status, patient location, and quarter of visit remained largely comparable across groups. The one notable difference was in ED disposition, where excluded cases had a higher proportion of admitted or transferred encounters (16% vs. 8.3%), likely reflecting the 32 in-hospital deaths that were removed because death as an ED disposition fell outside the binary outcome definition used in this analysis.

Given the overall similarity between included and excluded cases across key characteristics, the risk of selection bias introduced by complete-case analysis is considered low, though it cannot be entirely ruled out. That said, complete-case analysis may introduce bias if the probability of missingness is associated with factors related to emergency department disposition. Although excluded observations differed modestly from the analytic sample, the administrative structure of NEDS provides limited auxiliary information for reliably modeling missingness. Accordingly, these findings should be interpreted with appropriate caution. See Supplemental Table S2 for more information.

Fourth, the analysis is limited to a single calendar year, which precludes assessment of year-over-year trends. Although previous studies indicate that injury mechanisms and demographics in 2021 closely resembled those of earlier years, the 2021 data were collected during the ongoing pandemic recovery. This context may have caused residual distortions that extend beyond what injury mechanism trends alone can reflect. Reduced motor vehicle traffic volumes during this period could have altered the exposure context for motor vehicle-involved crashes, potentially affecting the observed patterns. Shifts in healthcare-seeking behavior and ED utilization patterns during pandemic recovery may similarly have influenced who presented to the ED and how disposition decisions were made. These pandemic-era contextual factors should be considered when interpreting the generalizability of the present findings to more recent years. The micromobility technology landscape has also continued to evolve since 2021, with shared fleet e-scooters increasingly featuring geofenced speed limiters and improved braking systems. Findings should therefore be interpreted as characteristic of a transitional period in micromobility growth rather than as a static characterization of current e-scooter injury risk.

Fifth, residual confounding remains possible because NEDS does not capture several factors that may influence injury severity and subsequent disposition, including helmet use, rider experience, vehicle speed, roadway characteristics, emergency medical services triage, trip- or mile-level exposure data, and physiologic indicators of injury severity. This absence limits the ability to characterize crash circumstances in detail and precludes any inference regarding which mode carries greater absolute riding risk. Therefore, the findings should be interpreted as reflecting injury and utilization patterns among those who sought emergency care rather than as a comparison of the inherent danger of each mode. Separately, substance use data are derived from diagnostic codes rather than toxicology results, increasing the likelihood of underreporting and potentially attenuating the estimated interaction with device type. Sixth, although grouping admission and transfer outcomes simplified the analysis, these represent potentially distinct clinical pathways that may obscure meaningful differences in patient management, such as short-stay observation versus transfer to a higher level of care.

Finally, the dataset captures only the initial ED visit and does not allow follow-up after discharge, so outcomes such as readmission, delayed complications, or post-discharge mortality cannot be assessed. Despite these limitations, this study provides a rigorous national framework for examining patterns of utilization of micromobility injuries and highlights opportunities for methodological refinement as more granular variables and linked pre-hospital and inpatient data become available.

## 5. Public Health Implications

The growing popularity of e-scooters as a short-distance urban transportation option presents both opportunities and challenges for injury prevention, healthcare preparedness, and transportation policy. While the present cross-sectional design cannot determine which specific interventions would be most effective, the observed injury and utilization patterns, considered alongside prior literature, suggest that mode-specific safety strategies merit serious attention. For e-scooter users, strategies worth evaluating include encouraging wrist and ankle protection, regulating sidewalk speeds, and improving pavement conditions to reduce the risk of low-energy falls. For bicyclists, policies aimed at minimizing collision risk and blunt-force trauma through expanded protected bike lanes and traffic-calming infrastructure remain well-supported by existing evidence.

From a policy perspective, the fragmentation of e-scooter regulation across state and municipal jurisdictions remains a significant public health concern. There are currently no consistent nationwide standards in the United States regarding minimum age, helmet use, riding while intoxicated, or permissible riding environments, resulting in an inconsistent regulatory landscape for riders [9,38,46,47]. In contrast, bicycles have benefited from decades of systematic safety research, school-based helmet promotion, legislative mandates, and infrastructure investment, all of which are associated with measurable reductions in head injuries over time [45,48,49]. The present findings, alongside prior evidence, suggest that analogous strategies, including age-specific helmet mandates, DUI enforcement, speed restrictions, and infrastructure protections, warrant consideration in the e-scooter context [25,46,50]. The adoption of mandatory helmet legislation and e-scooter-specific regulations in countries including Australia, New Zealand, Canada, and China demonstrates that such frameworks are feasible and have been pursued internationally in recognition of the injury risk associated with micromobility [51].

Public health plays an important role in reducing e-scooter injuries. Surveillance systems such as NEDS are valuable for documenting injury and utilization patterns, but standardized real-time monitoring would strengthen the ability to evaluate policy impact and track exposure trends over time. Standardizing the use of updated ICD-10-CM codes for e-scooter injuries is an essential step toward improving the consistency of surveillance and enabling the evaluation of safety interventions across jurisdictions. Education and awareness campaigns also merit investment, particularly for adolescents and first-time riders who are disproportionately represented in injury statistics [8,37,52]. Collaborations between public health agencies and e-scooter companies could expand access to helmets, embed in-app safety prompts, and promote sober, responsible riding. Industry-led initiatives such as Lime’s Respect the Ride campaign, which distributed helmets and promoted rider safety pledges, represent one documented example of this type of partnership [53].

Taken together, these findings underscore the importance of advancing e-scooter policy and practice in step with the rapid growth of micromobility. Decades of bicycle injury prevention research offer a potentially instructive framework for developing evidence-based e-scooter safety strategies, though the distinct infrastructure context, regulatory environment, and rider demographics of e-scooter use mean that direct translation of bicycle policies will require careful adaptation. Sustained collaboration among public health professionals, transportation planners, and city officials, integrating health, urban planning, and infrastructure perspectives, will be essential to fostering a culture of safety for all micromobility users.

## 6. Conclusions

In this national analysis of ED visits from the 2021 NEDS, e-scooter and bicycle injuries were associated with comparable overall odds of hospital admission or transfer after adjustment for demographic, temporal, and geographic factors. Nevertheless, motor vehicle involvement emerged as a critical effect modifier, with e-scooter riders experiencing significantly higher odds of admission or transfer than bicyclists in such encounters, underscoring the disproportionate healthcare resource utilization associated with vehicle-involved e-scooter crashes and identifying this subgroup as a high-priority target for clinical and policy intervention. Injury profile comparisons further revealed distinct biomechanical profiles between modes, with e-scooter encounters predominantly characterized by distal extremity injuries consistent with low-energy falls, and bicycle encounters more often involving proximal fractures and internal trauma suggestive of higher-energy impacts.

These findings carry meaningful implications for injury prevention and public health practice. The mode-specific injury patterns observed here suggest that safety strategies should be tailored accordingly, with e-scooter interventions prioritizing fall prevention and extremity protection and bicycle safety efforts focusing on infrastructure solutions that reduce exposure to high-energy collisions. At the policy level, the continued fragmentation of e-scooter regulation across state and municipal jurisdictions, in the absence of consistent federal standards, represents an ongoing public health concern that the present findings reinforce. Standardizing ICD-10-CM coding practices for e-scooter injuries and investing in real-time surveillance infrastructure would substantially improve the ability to monitor trends, evaluate policy impact, and identify high-risk subgroups over time. As micromobility ridership continues to grow and vehicle technology evolves, sustained collaboration among public health professionals, transportation planners, and city officials will be essential to translating emerging evidence into effective, equitable safety strategies for all riders.

## Figures and Tables

**Table 1:** Weighted Sample Characteristics of ED Encounters Involving Bicycles and E-scooters, 2021 Nationwide Emergency Department Sample.

Characteristic	Overall *n* = 239,457 ^1^	Bicycle *n* = 218,469 ^1^	E-Scooter *n* = 20,988 ^1^	*p*-Value ^2^
**ED Disposition**				0.007
Admitted/Transferred	19,515 (8.1%)	18,133 (8.3%)	1383 (6.6%)	
Treat and Release	219,942 (91.9%)	200,337 (91.7%)	19,605 (93.4%)	
**Age Categories**				<0.001
<18	91,786 (38.3%)	81,803 (37.4%)	9983 (47.6%)	
18–25	26,902 (11.2%)	23,937 (11.0%)	2966 (14.1%)	
26–40	50,829 (21.2%)	46,081 (21.1%)	4748 (22.6%)	
41–64	69,940 (29.2%)	66,649 (30.5%)	3291 (15.7%)	
**Race**				<0.001
White	138,548 (57.9%)	127,180 (58.2%)	11,369 (54.2%)	
Black	25,321 (10.6%)	22,171 (10.1%)	3149 (15.0%)	
Hispanic	44,676 (18.7%)	40,896 (18.7%)	3779 (18.0%)	
Asian or Pacific Islander	9693 (4.0%)	8914 (4.1%)	779 (3.7%)	
Other	13,631 (5.7%)	12,462 (5.7%)	1168 (5.6%)	
Unknown	7589 (3.2%)	6845 (3.1%)	743 (3.5%)	
**Sex**				<0.001
Female	63,731 (26.6%)	53,974 (24.7%)	9757 (46.5%)	
Male	175,726 (73.4%)	164,495 (75.3%)	11,231 (53.5%)	
**Household Income Quartile**				<0.001
$1–$51,999	55,575 (23.4%)	49,383 (22.8%)	6192 (29.7%)	
$52,000–$65,999	54,870 (23.1%)	49,637 (22.9%)	5234 (25.1%)	
$66,000–$87,999	58,107 (24.5%)	53,403 (24.7%)	4704 (22.6%)	
$88,000+	68,625 (28.9%)	63,909 (29.5%)	4715 (22.6%)	
Unknown	2280 (0.1%)	2137 (0.1%)	143 (0.0%)	
**Primary Insurance**				<0.001
No Insurance/Under-insured	36,008 (15.0%)	33,243 (15.2%)	2764 (13.2%)	
Public	90,368 (37.7%)	81,236 (37.2%)	9132 (43.5%)	
Private	113,082 (47.2%)	103,990 (47.6%)	9091 (43.3%)	
**Motor Vehicle Involved**				<0.001
No	200,811 (83.9%)	181,440 (83.1%)	19,371 (92.3%)	
Yes	38,646 (16.1%)	37,030 (16.9%)	1616 (7.7%)	
**Substance Use**				0.2
No Substance use	210,918 (88.1%)	192,264 (88.0%)	18,654 (88.9%)	
Alcohol	125 (0.1%)	105 (0.0%)	20 (0.1%)	
Drugs	25,929 (10.8%)	23,862 (10.9%)	2067 (9.8%)	
Both	2485 (1.0%)	2237 (1.0%)	247 (1.2%)	
**Quarter of Visit**				<0.001
1	37,251 (15.6%)	33,268 (15.2%)	3983 (19.0%)	
2	79,047 (33.0%)	72,785 (33.3%)	6262 (29.8%)	
3	80,652 (33.7%)	74,733 (34.2%)	5919 (28.2%)	
4	42,507 (17.8%)	37,683 (17.2%)	4824 (23.0%)	
**Weekend**				<0.001
No	157,783 (65.9%)	144,535 (66.2%)	13,248 (63.1%)	
Yes	81,674 (34.1%)	73,934 (33.8%)	7740 (36.9%)	
**Patient Location**				0.3
Central metro ≥ 1 M	86,341 (36.1%)	78,747 (36.0%)	7593 (36.2%)	
Fringe metro ≥ 1 M	53,123 (22.2%)	48,639 (22.3%)	4484 (21.4%)	
Metro 250 k–999 k	47,403 (19.8%)	43,419 (19.9%)	3984 (19.0%)	
Metro 50 k–249 k	20,391 (8.5%)	18,367 (8.4%)	2024 (9.6%)	
Micropolitan	20,958 (8.8%)	19,165 (8.8%)	1793 (8.5%)	
Non-core (rural)	11,242 (4.7%)	10,132 (4.6%)	1109 (5.3%)	
**Hospital Region**				<0.001
Midwest	43,281 (18.1%)	38,773 (17.7%)	4508 (21.5%)	
Northeast	56,007 (23.4%)	52,043 (23.8%)	3964 (18.9%)	
South	58,202 (24.3%)	52,149 (23.9%)	6053 (28.8%)	
West	81,967 (34.2%)	75,505 (34.6%)	6462 (30.8%)	
**Hospital Control**				0.4
Government or Private (collapsed)	37,680 (15.7%)	34,629 (15.9%)	3051 (14.5%)	
Government, nonfederal (public)	32,706 (13.7%)	29,382 (13.4%)	3324 (15.8%)	
Private (collapsed)	34,588 (14.4%)	31,451 (14.4%)	3137 (14.9%)	
Private, investor-owned (proprietary)	22,885 (9.6%)	20,803 (9.5%)	2082 (9.9%)	
Private, not-for-profit (voluntary)	111,598 (46.6%)	102,204 (46.8%)	9394 (44.8%)	
**Hospital Trauma Level**				0.004
Not a trauma center	114,591 (48.3%)	104,649 (48.4%)	9942 (47.6%)	
Trauma center level I	52,025 (21.9%)	46,528 (21.5%)	5497 (26.3%)	
Trauma center level II	41,505 (17.5%)	38,644 (17.9%)	2861 (13.7%)	
Trauma center level III	29,101 (12.3%)	26,531 (12.3%)	2570 (12.3%)	
Unknown	2234 (0.0%)	2117 (0.0%)	117 (0.0%)	
**Hospital Teaching Status**				0.13
Non-metropolitan hospital	33,339 (13.9%)	30,753 (14.1%)	2586 (12.3%)	
Metropolitan non-teaching	46,089 (19.2%)	42,237 (19.3%)	3852 (18.4%)	
Metropolitan teaching	160,029 (66.8%)	145,479 (66.6%)	14,550 (69.3%)	

^1^ Weighted frequency; percentages are survey weighted. ^2^ Pearson’s X^2^: Rao & Scott adjustment. Weighted counts within variable categories may not sum exactly to the total due to rounding of survey-weighted estimates. Missing values for household income quartile (*n* = 2280) and hospital trauma level (*n* = 2234) reflect suppressed or unreported data in the 2021 NEDS and were not used in regression analyses.

**Table 2:** Weighted Summary of Injuries Sustained Among Bicyclists and E-scooter Riders, 2021 Nationwide Emergency Department Sample.

Characteristic	Overall *n* = 239,457 ^1^	Bicycle *n* = 218,469 ^1^	E-Scooter *n* = 20,988 ^1^	*p*-Value ^2^
Minor Head Injury				0.2
	32,406 (13.5%)	29,698 (13.6%)	2709 (12.9%)	
Intracranial Injury				0.5
	16,784 (7.0%)	15,386 (7.0%)	1398 (6.7%)	
Proximal Upper Extremity Fractures				<0.001
	21,864 (9.1%)	20,259 (9.3%)	1605 (7.6%)	
Distal Upper Extremity Fractures				<0.001
	35,472 (14.8%)	30,641 (14.0%)	4831 (23.0%)	
Proximal Lower Extremity Fractures				0.040
	2511 (1.0%)	2351 (1.1%)	160 (0.8%)	
Distal Lower Extremity Fractures				0.001
	11,505 (4.8%)	10,254 (4.7%)	1251 (6.0%)	
Fractures of Skull and Facial Bones				0.4
	10,314 (4.3%)	9473 (4.3%)	841 (4.0%)	
Fractures of Pelvis and Lumbar Spine				<0.001
	3461 (1.4%)	3308 (1.5%)	153 (0.7%)	
Fractures of Ribs, Sternum, and Thoracic Spine				<0.001
	11,301 (4.7%)	10,922 (5.0%)	379 (1.8%)	
Dislocations				<0.001
	8775 (3.7%)	8298 (3.8%)	477 (2.3%)	
Sprains				0.007
	16,305 (6.8%)	14,670 (6.7%)	1635 (7.8%)	
Intra-abdominal or Intrathoracic Injury				<0.001
	7623 (3.2%)	7333 (3.4%)	290 (1.4%)	
Lacerations				<0.001
	50,638 (21.1%)	47,239 (21.6%)	3399 (16.2%)	

^1^ Weighted frequency; percentages are survey weighted. ^2^ Pearson’s X^2^: Rao & Scott adjustment. Percentages reflect the proportion of encounters in which each injury type was present and do not sum to 100% because patients may sustain multiple injuries within a single ED encounter.

**Table 3:** Association Between Mode of Transportation (E-scooter vs. Bicycle) and ED Disposition (Hospital Admission or Transfer vs. Treat-and-Release), Overall and Stratified by Motor-Vehicle Involvement—2021 NEDS.

Model	Type	OR (95% CI)
Overall		
	Unadjusted	0.78 (0.65, 0.94)
	Fully Adjusted ^1^	1.03 (0.86, 1.23)
Motor Vehicle Involved		
No	Fully Adjusted ^1^	0.99 (0.83, 1.16)
Yes	Fully Adjusted ^1^	1.67 (1.12, 2.48)
	Bicycle *n* = 218,469 ^2^	E-Scooter *n* = 20,988 ^2^
Motor Vehicle Involved		
No	42,593 (83.1%)	4605 (92.3%)
Yes	8791 (16.9%)	365 (7.7%)

^1^ Fully adjusted models controlled for age, sex, race, quarter, weekend indicator, patient location, hospital region, substance use, motor-vehicle involvement, and primary insurance type. Stratified estimates were derived from an interaction model between mode of transportation and motor-vehicle involvement. ^2^ Unweighted frequency; percentages are survey weighted.

## Data Availability

Restrictions apply to the availability of these data. Data were obtained from the Healthcare Cost and Utilization Project (HCUP) Nationwide Emergency Department Sample (NEDS) and are available from https://hcup-us.ahrq.gov/ (accessed on 5 July 2026) upon completion of the required training and execution of a signed Data Use Agreement.

## Supplementary Materials

The following supporting information can be downloaded at https://www.mdpi.com/article/10.3390/ijerph23080972/s1, Supplemental Table S1: ICD-10 diagnosis and procedure codes for identification of mode of transportation, effect modifiers, and injuries associated with the study population; Supplemental Table S2: Characteristics of participants who had ED Disposition present and were excluded from the analytic sample.
