# Supplementary material for: E-Scooter and Bicycle Healthcare Utilization in U.S. Emergency Departments: Examined Through Mode-Specific Diagnostic Codes
Source: Int J Environ Res Public Health. 2026 Jul 28;23(8):972. doi: 10.3390/ijerph23080972 (PMC13513584; doi:10.3390/ijerph23080972)
Supplement: Supplementary file 1 [file ijerph-23-00972-s001.zip › Supplemental Table S1 Decsription ICD 10 Diagnostics Codes.pdf]

Supplemental Table S1: ICD-10 diagnosis and procedure codes for identification of mode of transportation, effect modifiers, and injuries associated with the study population.

Characteristic	ICD-10 diagnosis code	Diagnosis description
**Mode of Transportation**		
Conventional Bicycle	V10-19.-	Collisions that involve a pedal cycle
Electric Scooter	V00.84-,<br><br>V01.031-, V01.131-, V01.931-, V02.031-, V02.131-, V02.931-, V03.031-, V03.131-, V03.931-, V04.031-, V04.131-, V04.931-, V05.031-, V05.131-, V05.931-, V06.031-, V06.131-, V06.931-	Standing electric scooters encounter Pedestrians, resulting in collisions between pedal cycles, motor vehicles, or trains.
**Effect Modifiers**		
Motor vehicle + e-scooter	V03.031-, V03.131-, V03.931-, V04.031-, V04.131-, V04.931-, V05.031-, V05.131-, V05.931-,	Collisions between electric scooters and a motor vehicle or train.
Motor vehicle + Bicycle	V12-15.5-, V19.0-2, V19.4-8-	Collision between a pedal cycle and a motor vehicle or train.
Alcohol Use	F10.-, R78.0, Y90.0-9	Alcohol use, finding Alcohol in Blood, and associated blood alcohol level.
Drug Use	F11–19-, R78.1-6-	Use of opiates, cocaine, hallucinogens, or other steroid agents in the blood.
**Injuries and Medical Conditions**		
Minor Head Injury	S00.0-7	Superficial injury of the head
	S01.00-05	Open wound of the head
Intracranial Injury	S06.0-9, A	
Proximal Upper Extremity Fractures	S42.0-9	Fractures of the clavicle, scapula, humerus, and shoulder girdle.
Distal Upper Extremity Fractures	S52.0-9	Fractures of the ulna and radius.
	S62.0-9	Fractures of the wrist, carpal, and metacarpal bones.
Proximal Lower Extremity Fractures	S72.0-9	Fractures of the femur
Distal Lower Extremity Fractures	S82.0-9	Fractures of the patella, tibia, fibula, and malleoli
	S92.0-9	Fractures of calcaneus, talus, tarsal, and metatarsal bones

Characteristic	ICD-10 diagnosis code	Diagnosis description
Fractures of the skull and facial bones	S02.0-9	Fractures of the vault/base of skull, nasal bones, orbital floor, malar, mandibular, and other
Fractures of the pelvis and lumbar spine	S32.0-9	Fractures of the sacrum, coccyx, ilium, acetabulum, pubis, ischium, and lumbar spine
Fractures of ribs, sternum, and thoracic spine	S22.0-9	Fractures of the sternum, ribs, and thoracic spine
Dislocations	S43.0-39, S53.0-53.19, S63.1-2, S73.0-04, S83.0-1, S63.0, S93.0-06, S93.1-3	Dislocation of the shoulder, acromioclavicular, elbow, fingers, hip, knee, wrist, ankle, and foot joints
Sprains	S43.4-9,53.4,63.5-9, 73.1,83.4-9,93.4	Sprain of joints and ligaments of the shoulder girdle, elbow, wrist, fingers, hip, knee, ankle, foot, and toe
Intra-abdominal or intrathoracic injuries	S26-29, SS36, S37	Injury to the heart, thorax, abdominal organs, urinary and pelvic organs
Lacerations	S01,11,21,31,41,51,61,71,81,91	Lacerations to the head, neck, thorax, abdomen, back, shoulder, elbow, wrist, hand, fingers, hip, thigh, knee, and lower leg.
Neurological Disorders		
	G00-G09	Inflammatory diseases of the central nervous system
	G10-G14	Systemic atrophies primarily affect the central nervous system.
	G20-G26	Extrapyramidal and movement disorders
	G30-G32	Other degenerative diseases of the nervous system
	G35-G37	Demyelinating diseases of the central nervous system
	G40-G47	Episodic and paroxysmal disorders
	G50-G59	Nerve, nerve root, and plexus disorders
	G60-G65	Polyneuropathies and other disorders of the peripheral nervous system
	G70-G73	Diseases of the myoneural junction and muscle
	G80-83	Cerebral palsy and other paralytic syndromes
	G89-G99	Other disorders of the nervous system

ICD-10: International Classification of Diseases, 10th Revision.
