# Supplementary material for: E-Scooter and Bicycle Healthcare Utilization in U.S. Emergency Departments: Examined Through Mode-Specific Diagnostic Codes
Source: Int J Environ Res Public Health. 2026 Jul 28;23(8):972. doi: 10.3390/ijerph23080972 (PMC13513584; doi:10.3390/ijerph23080972)
Supplement: Supplementary file 1 [file ijerph-23-00972-s001.zip › Supplemental Table S2 Characteristics of Excluded Participants.pdf]

Supplemental Table S2: Characteristics of participants who had ED Disposition present and were excluded from the analytic sample

Characteristic	Overall n = 57,376 [1]	Included n = 56,354 [1]	Excluded n = 1,022 [1]
**Exposure**			
Bicycle	52,367 (91%)	51,384 (91%)	983 (96%)
E-scooter	5,009 (8.7%)	4,970 (8.8%)	39 (3.8%)
**Outcome**			
Admitted/Transferred	4,818 (8.4%)	4,664 (8.3%)	154 (16%)
Treat and Release	52,526 (92%)	51,690 (92%)	836 (84%)
Died	32	0	32
**Sex**			
Female	15,158 (26%)	14,984 (27%)	174 (17%)
Male	42,201 (74%)	41,370 (73%)	831 (83%)
Unknown	17	0	17
**Primary Insurance**			
No Insurance/ Under-insured	8,927 (16%)	8,656 (15%)	271 (30%)
Public	22,023 (38%)	21,539 (38%)	484 (54%)
Private	26,298 (46%)	26,159 (46%)	139 (16%)
Unknown	128	0	128
**Patient Location**			
Central metro ≥1M	20,018 (35%)	19,955 (35%)	63 (36%)
Fringe metro ≥1M	12,401 (22%)	12,382 (22%)	19 (11%)
Metro 250k–999k	12,090 (21%)	12,054 (21%)	36 (21%)
Metro 50k–249k	4,910 (8.7%)	4,901 (8.7%)	9 (5.2%)
Micropolitan	4,451 (7.9%)	4,422 (7.8%)	29 (17%)
Non-core (rural)	2,658 (4.7%)	2,640 (4.7%)	18 (10%)
Unknown	848	0	848
**Quarter of Visit**			
1	9,290 (16%)	9,116 (16%)	174 (18%)
2	18,812 (33%)	18,564 (33%)	248 (26%)
3	18,788 (33%)	18,460 (33%)	328 (34%)
4	10,427 (18%)	10,214 (18%)	213 (22%)
Unknown	59	0	59

[1] Unweighted frequency; percentages are unweighted.
